# Various GLP-1 Receptor Agonist Preference Use with a Special Focus on Oral and Subcutaneous Forms in Poland

**DOI:** 10.3390/healthcare13222874

**Published:** 2025-11-12

**Authors:** Klaudia Nowak, Artur Dziewierz, Aleksandra Sojda, Michał Zabojszcz, Łukasz Szarpak, Natalia Dardzinska, Paulina Jaskulska, Zbigniew Siudak

**Affiliations:** 1 Department of Internal Medicine and Cardiology, Collegium Medicum, Jan Kochanowski University, 19a, IX Wieków Kielc Street, 25-516 Kielce, Poland; kl.nowak@o2.pl (K.N.); alexandra.sojda@gmail.com (A.S.); michalzabojszcz@gmail.com (M.Z.); nataliad0609@icloud.com (N.D.); paulina672@gmail.com (P.J.); 2 Collegium Medicum, Jagiellonian University in Krakow, 31-008 Krakow, Poland; artur.dziewierz@uj.edu.pl; 3 Department of Clinical Research and Development, LUXMED Group, 02-676 Warsaw, Poland; lukasz.szarpak@gmail.com; 4 Institute of Medical Science, Collegium Medicum, The John Paul II Catholic University of Lublin, 20-400 Lublin, Poland; 5 Henry JN Taub Department of Emergency Medicine, Baylor College of Medicine, Houston, TX 77030, USA

**Keywords:** obesity, diabetes mellitus type 2, GLP-1 receptor agonists

## Abstract

**Background**: Since the introduction of the first GLP-1 receptor agonist (GLP-1 RA) in 2005, there has been a steady increase in the number of drugs available in this group, as well as an expansion of their indications and routes of administration. **Aim**: The aim of the study was to assess the clinical characteristics of patients treated with GLP-1 RA in Poland in 2018–2024, with particular emphasis on the disease entities constituting indications for treatment (like obesity and diabetes), and to analyse the frequency of use of individual drugs during the study period. **Methods**: A cohort study was conducted based on anonymised medical data from 300 outpatient clinics the largest private healthcare facilities in Poland (Luxmed), on consecutive patients who had at least one prescription for GLP-1 RA. The analysis covered the period from 1 January 2018 to 31 December 2024. **Results**: The number of patients using GLP-1 RA increased from 212 in 2018 to 12,836 in 2024. Obesity was diagnosed in 78% of all patients, most often in the groups using liraglutide and tirzepatide. The highest percentage of patients with type 2 diabetes was observed in the dulaglutide group (67%), while the lowest was in the tirzepatide group (15%). From 2022, the share of oral semaglutide steadily increased, reaching 50% of all semaglutide applications in 2024 in Poland. **Conclusions**: In the analysed group, GLP-1 RAs were most commonly used to treat obesity. The oral form of semaglutide was more frequently used in younger females with less aggravating medical history.

## 1. Introduction

The first GLP-1 receptor agonist (GLP-1 RA) approved by the FDA was exenatide, which was authorised for use in 2005. Since then, new drugs have appeared on the market, starting with the introduction of liraglutide in 2010, dulaglutide in 2014, and semaglutide in 2017. Over the years, their indications for use have expanded—initially, they were recommended only for the treatment of type 2 diabetes. The route of administration of the drugs has also improved, as initially all drugs were administered subcutaneously, while semaglutide is now also available in oral form [1]. The first GLP-1 RA registered for the treatment of obesity was liraglutide (2014), followed by semaglutide s.c. (2021) and tirzepatide (2023). GLP-1 analogues contribute to weight loss and carbohydrate metabolism balance in patients by regulating glucose levels through stimulating glucose-dependent insulin secretion, slowing gastric emptying, and increasing feelings of satiety. In addition, the dual GLP-1/GIP (glucose-dependent insulinotropic polypeptide) agonist tirzepatide further suppresses appetite by signalling receptors in brain cells. The SURMOUNT series of studies (1–5) showed that tirzepatide led to an average weight loss of −20.9% at the highest dose of 15 mg (*p* < 0.001) [2,3]. In addition, tirzepatide was found to be superior to semaglutide in reducing waist circumference and body weight. Over 72 weeks, obese patients without type 2 diabetes who were treated with tirzepatide achieved a body weight reduction of −20.9%, while those treated with semaglutide achieved a reduction of 13.7% [4]. In addition to controlling body weight and blood glucose levels, GLP-1 analogues also have other beneficial effects, such as reducing inflammation and postprandial lipemia and lowering blood pressure. The SURPASS clinical trial showed that tirzepatide reduces the incidence of metabolic syndrome, highlighting the cardiovascular benefits of its use [1].

Of current GLP-1 RAs, the following are available in Poland: semaglutide s.c./p.o., dulaglutide, liraglutide, and tirzepatide [5,6]. The indications for the use of semaglutide, liraglutide, and tirzepatide are a BMI ≥ 30 kg/m^2^ and a BMI ≥ 27 kg/m^2^ but <30 kg/m^2^ with obesity-related comorbidities. Tirzepatide is also indicated for inadequately controlled type 2 diabetes. Dulaglutide and semaglutide p.o. are only registered for the treatment of type 2 diabetes in adults [5,6]. Two preparations are reimbursed in Poland: semaglutide s.c. and dulaglutide in patients with type 2 diabetes treated with at least two hypoglycaemic drugs, with HbA1c ≥ 7.5%, obesity, defined as BMI ≥ 30 kg/m^2^, and very high cardiovascular risk, which is defined as confirmed cardiovascular disease, damage to other organs manifested by proteinuria or left ventricular hypertrophy or retinopathy, or the presence of at least two major risk factors among those listed (age ≥ 55 years for men, ≥60 years for women, dyslipidaemia, hypertension, and smoking [5,6]. Between 2014 and 2022, the number of people taking GLP-1-RA increased rapidly—in Australia, it increased tenfold [7]. This trend is also observed in Poland [8,9,10,11].

## 2. Materials and Methods

This was a retrospective cohort study using blinded data from Luxmed, which is a major private healthcare provider in Poland, from 1 January 2018 till 31 December 2024. A detailed description of the study database has been provided in previous papers [8,9,10,11]. Patients in this analysis included individuals ≥18 years who were prescribed a GLP-RA or GLP-1 RA/GIP (glucose-dependent insulinotropic polypeptide) for any indication (diabetes, obesity, or off-label). There were no exclusion criteria. Individual unique patient data are reported. This retrospective cohort study analysed anonymised data from an electronic health record (EHR) system, covering 300 outpatient clinics across Poland. GLP-1 RA and dual GIP/GLP-1 RA prescriptions were thus identified from physician-generated EHR prescriptions representing prescribed medications. Demographics (age, sex) were determined at the index. Clinical characteristics (comorbidities) were ascertained from the 24-month pre-index period. Comorbidities were flagged as present if diagnostic codes appeared at least once during the 24-month lookback window. The study received approval from the Bioethics Committee at Jan Kochanowski University in Kielce, Poland (No. 62/2024 of 13 November 2024), and was conducted in accordance with the Declaration of Helsinki. Patient consent was waived due to the retrospective, anonymised nature of the data.

### Statistical Analysis

Categorical variables were presented as counts with percentages, and continuous variables as medians with interquartile ranges. Group comparisons were performed using the Kruskal–Wallis rank sum test, Pearson’s Chi-squared test, and Fisher’s exact test, where applicable. A two-tailed *p*-value < 0.05 was considered statistically significant. All statistical analyses were performed by an experienced statistician using IBM SPSS Statistics version 29.0.2.0 (IBM Corp., Armonk, NY, USA).

## 3. Aim of the Study

The aim of the study was to assess the clinical characteristics of patients receiving GLP-1 RA between 2018, when they first became available, and 2024 in Poland, with particular emphasis on the disease entities constituting an indication for therapy. In addition, an analysis of the frequency of use of individual drugs during the study period was performed, allowing therapeutic trends in the analysed population to be identified.

## 4. Results

The study involved 36,399 patients from Poland. Of these, 4442 (12.2%) were prescribed dulaglutide, 13,298 (36.5%) liraglutide, 16,227 (44.6%) semaglutide, and 2432 (6.7%) tirzepatide. Women constituted the majority of the study population—25,192 (69%), which was evident in all drug subgroups and differed significantly between GLP-1 RA types (*p* < 0.001). The demographic and clinical characteristics of participants are shown in Table 1. Specific distinctions between populations of patients receiving oral and subcutaneous forms of semaglutide are presented in Table 2.

The frequency of GLP-1 RA use increased steadily between 2018 and 2024, from 212 patients in 2018 to 12,836 patients in 2024. Initially, only dulaglutide and liraglutide were available; semaglutide in subcutaneous form appeared in 2020, in oral form in 2022, and tirzepatide in 2024. During the analysed period, the most commonly used drug was liraglutide (n = 13,298; 37%), and the least frequently used was tirzepatide (n = 2432; 6.7%) due to its late availability in Poland. Although oral semaglutide has been used in our patients since 2022, in 2024, it accounted for 50% of all semaglutides used. Detailed characteristics of the use of each drug by year are shown in Table 3.

Additional analysis revealed that the temporal changes were significant both for men and women, as seen in Table 4 and Table 5, as well as for patients younger than and older than 46 years (the median age in our cohort), as presented in Table 6 and Table 7. A significant decrease in the use of dulaglutide and liraglutide was observed from 2018 to 2024 in obese patients (Table 8).

## 5. Discussion

The presented results confirm the global trend of systematic growth in the use of GLP-1 analogues. Similarly to the analysis by Watanabe et al., in which the number of patients using semaglutide increased from 569 in 2019 to 22,891 in 2022, in Poland, between 2018 and 2024, we also recorded a significant increase in the number of patients receiving this group of drugs—from 212 in 2018 to 12,836 in 2024 [12].

A particularly interesting observation is the fact that in Poland, semaglutide and dulaglutide are reimbursed for the treatment of type 2 diabetes, but in the analysed population, a significant percentage of patients using these preparations did not have diagnosed diabetes. Type 2 diabetes was present in only 41% of patients taking semaglutide s.c. and 67% of those treated with dulaglutide, while obesity was found in 76% and 71% of patients, respectively. However, our results show that a significant proportion of patients taking these drugs did not have a documented diagnosis of diabetes, which may reflect their use in the treatment of obesity. These results may indicate the growing role of GLP-1 RA beyond the classic diabetic indication. A similar trend was observed for semaglutide p.o., formally registered only for the treatment of type 2 diabetes but used in patients, 74% of whom were obese, while only 35% had type 2 diabetes.

In the case of liraglutide, its form intended for the treatment of obesity was clearly preferred over the other form, indicated for type 2 diabetes, as 84% of patients taking liraglutide were obese. These data confirm that in Polish clinical practice, GLP-1 RAs are increasingly used in the pharmacotherapy of obesity, which is consistent with current international guidelines recommending the use of liraglutide, semaglutide, and tirzepatide in the treatment of obesity [13].

In recent years, the beneficial effects of GLP-1 RAs have been increasingly emphasised, not only in terms of weight reduction and glycaemic control, but also in terms of obstructive sleep apnoea. Clinical trials have shown that these drugs effectively reduce the apnoea-hypopnoea index (AHI), with tirzepatide showing the greatest efficacy and being more effective in obese individuals [14]. In December 2024, the FDA (Food and Drug Administration) approved tirzepatide for the treatment of moderate to severe obstructive sleep apnoea in obese adults in combination with a low-calorie diet and increased physical activity [15]. In our analysis, sleep apnoea was diagnosed in 1899 patients (5.2%), which highlights the importance of this condition in the population of people using GLP-1 RA.

Of our 945 patients, 945 had chronic kidney disease, with the highest incidence occurring in the dulaglutide group (6.1%; *p* < 0.001). According to the latest KDIGO 2024 guidelines, the use of GLP-1 RAs is recommended in chronic kidney disease. The FLOW study showed that the use of GLP-1 RAs in chronic kidney disease, type 2 diabetes, and albuminuria reduces the incidence of kidney-related adverse events [16].

In the analysed population, tumours of uncertain or unknown endocrine origin were very rare—only one case was observed among patients treated with subcutaneous semaglutide. The possible relationship between GLP-1 RAs and cancer risk, especially thyroid cancer, is still unclear. According to Brito et al., GLP-1 RAs do not affect the incidence of most malignant tumours and may reduce the risk of cancers commonly associated with obesity. However, in long-term observations, the authors reported a possible increase in the risk of thyroid cancer [17]. In contrast, the meta-analysis by Baxter et al. found no evidence of a higher thyroid cancer risk during short-term GLP-1 RA use [18]. While the use of tirzepatide has sharply increased in Poland, its high out-of-pocket cost and lack of reimbursement often lead to premature discontinuation and lack of clinical efficacy, which are not as evident in other GLP-1 RAs used in Poland [11,19,20].

## 6. Conclusions

We have noticed significant differences between the registration indications for various GLP-1 RA and their use in clinical practice in Poland in recent years. Dulaglutide, intended exclusively for the treatment of type 2 diabetes, was also used in patients with obesity, while liraglutide, semaglutide, and tirzepatide, even though registered for both diabetes and obesity treatment, were primarily utilised for weight loss management. These results indicate that, regardless of registration differences, obesity was the dominant area of application for these drugs in everyday medical practice. The oral form of semaglutide was more frequently used in younger females with less aggravating medical history.

## Figures and Tables

**Table 1:** Demographic and clinical characteristics of participants (*p*-value for all in-between GLP-1 RA comparisons).

Characteristic	Overall	Dulaglutide	Liraglutide	Semaglutide	Tirzepatide	*p*-Value
Number of Participants	36,399	4442	13,298	16,227	2432	
Age Median (Q1–Q3)	46	54	42	48	44	<0.001
	(37–56)	(45–65)	(35–50)	(39–58)	(36–52)	
Age Mean (SD)	47 (13)	55 (13)	43 (12)	49 (13)	44 (11)	<0.001
Age Min/Max	18/102	19/95	21/102	21/93	18/84	<0.001
**Gender**						
Women	25,192 (69%)	2363 (53%)	10,512 (79%)	10,691 (66%)	1626 (67%)	<0.001
Men	11,207 (31%)	2079 (47%)	2786 (21%)	5536 (34%)	806 (33%)	<0.001
**Number of participants with diseases taking specific medications**						
		**Dulaglutide**	**Liraglutide**	**Semaglutide**	**Tirzepatide**	***p*** **-value**
Diabetes Mellitus	11,315	2976 (67%)	1652 (12%)	6322 (39%)	365 (15%)	<0.001
Obesity (BMI > 30)	28,545	3175 (71%)	11,160 (84%)	12,164 (75%)	2046 (84%)	<0.001
Sleep Apnea	1899	304 (6.8%)	591 (4.4%)	890 (5.5%)	114 (4.7%)	<0.001
Dyslipidemias	14,825	2572 (58%)	4213 (32%)	7213 (44%)	827 (34%)	<0.001
Chronic Kidney Disease	945	272 (6.1%)	184 (1.4%)	456 (2.8%)	33 (1.4%)	<0.001

**Table 2:** Demographic and clinical characteristics of subcutaneous and oral semaglutide (*p*-value for all in-between GLP-1 RA comparisons).

Characteristic	Overall	Semaglutide p.o.	Semaglutide s.c.	*p*-Value
Number of Participants	16,227	5429	10,798	
Age Median (Q1–Q3)	48	47	48	<0.001
	(39–58)	(38–57)	(39–58)	
Age Mean (SD)	49 (13)	48 (13)	49 (12)	<0.001
Age Min/Max	18/93	14/89	18/93	<0.001
**Gender**				
Women	10,691 (66%)	3728 (69%)	6963 (64%)	<0.001
Men	5536 (34%)	1701 (31%)	3835 (36%)	<0.001
**Number of participants with diseases taking specific medications**				
		**Semaglutide p.o.**	**Semaglutide s.c.**	***p*** **-value**
Diabetes Mellitus	6322	1916 (35%)	4406 (41%)	<0.001
Obesity (BMI > 30)	12,164	4007 (74%)	8157 (76%)	<0.001
Sleep Apnea	890	273 (5.0%)	617 (5.7%)	0.070
Dyslipidemias	7213	2348 (43%)	4865 (45%)	0.029
Chronic Kidney Disease	456	119 (2.2%)	337 (3.1%)	<0.001

**Table 3:** Use of drugs according to year (*p*-value for all in-between GLP-1 RA comparisons).

Year	Overall	Number of Participants with Diseases Taking Specific Medications				
-	-	Dulaglutide	Liraglutide	Semaglutide p.o.	Semaglutide s.c.	Tirzepatide
2018–2024	36,399	4442 (12%)	13,298 (37%)	5429 (15%)	10,798 (30%)	2432 (6.7%)
2018	212	133 (63%)	79 (37%)	0 (0%)	0 (0%)	0 (0%)
2019	557	291 (52%)	266 (48%)	0 (0%)	0 (0%)	0 (0%)
2020	953	241 (25%)	437 (46%)	0 (0%)	275 (29%)	0 (0%)
2021	3081	535 (17%)	1227 (40%)	0 (0%)	1319 (43%)	0 (0%)
2022	7850	1716 (22%)	3445 (44%)	651 (8.3%)	2038 (26%)	0 (0%)
2023	10,910	899 (8.2%)	4183 (38%)	1703 (16%)	4125 (38%)	0 (0%)
2024	12,836	627 (4.9%)	3661 (29%)	3075 (24%)	3041 (24%)	2432 (19%)

**Table 4:** Use of various GLP-1 RAs according to year in females (*p*-value for all in-between GLP-1 RA comparisons).

Characteristic	N	Overall N = 25,192	2018 N = 92	2019 N = 319	2020 N = 633	2021 N = 2155	2022 N = 5651	2023 N = 7573	2024 N = 8769	*p*-Value
	25,192									<0.001
Dulaglutide		2363 (9.4%)	48 (52%)	141 (44%)	125 (20%)	292 (14%)	991 (18%)	463 (6.1%)	303 (3.5%)	
Liraglutide		10,512 (42%)	44 (48%)	178 (56%)	346 (55%)	994 (46%)	2830 (50%)	3254 (43%)	2866 (33%)	
Semaglutide p.o.		3728 (15%)	0 (0%)	0 (0%)	0 (0%)	0 (0%)	419 (7.4%)	1196 (16%)	2113 (24%)	
Semaglutide s.c.		6963 (28%)	0 (0%)	0 (0%)	162 (26%)	869 (40%)	1411 (25%)	2660 (35%)	1861 (21%)	
Tirzepatide		1626 (6.5%)	0 (0%)	0 (0%)	0 (0%)	0 (0%)	0 (0%)	0 (0%)	1626 (19%)	

**Table 5:** Use of various GLP-1 RAs according to year in males (*p*-value for all in-between GLP-1 RA comparisons).

Characteristic	N	Overall N = 11,207	2018 N = 120	2019 N = 238	2020 N = 320	2021 N = 926	2022 N = 2199	2023 N = 3337	2024 N = 4067	*p*-Value
	11,207									<0.001
Dulaglutide		2079 (19%)	85 (71%)	150 (63%)	116 (36%)	243 (26%)	725 (33%)	436 (13%)	324 (8.0%)	
Liraglutide		2786 (25%)	35 (29%)	88 (37%)	91 (28%)	233 (25%)	615 (28%)	929 (28%)	795 (20%)	
Semaglutide p.o.		1701 (15%)	0 (0%)	0 (0%)	0 (0%)	0 (0%)	232 (11%)	507 (15%)	962 (24%)	
Semaglutide s.c.		3835 (34%)	0 (0%)	0 (0%)	113 (35%)	450 (49%)	627 (29%)	1465 (44%)	1180 (29%)	
Tirzepatide		806 (7.2%)	0 (0%)	0 (0%)	0 (0%)	0 (0%)	0 (0%)	0 (0%)	806 (20%)	

**Table 6:** Use of various GLP-1 RAs according to year in patients younger than 46 years (*p*-value for all in-between GLP-1 RA comparisons).

Characteristic	N	Overall N = 18,678	2018 N = 33	2019 N = 146	2020 N = 352	2021 N = 1317	2022 N = 3958	2023 N = 5654	2024 N = 7218	*p*-Value
	18,678									<0.001
Dulaglutide		1263 (6.8%)	18 (55%)	60 (41%)	46 (13%)	146 (11%)	568 (14%)	241 (4.3%)	184 (2.5%)	
Liraglutide		8480 (45%)	15 (45%)	86 (59%)	223 (63%)	675 (51%)	2198 (56%)	2785 (49%)	2498 (35%)	
Semaglutide p.o.		2666 (14%)	0 (0%)	0 (0%)	0 (0%)	0 (0%)	289 (7.3%)	790 (14%)	1587 (22%)	
Semaglutide s.c.		4830 (26%)	0 (0%)	0 (0%)	83 (24%)	496 (38%)	903 (23%)	1838 (33%)	1510 (21%)	
Tirzepatide		1439 (7.7%)	0 (0%)	0 (0%)	0 (0%)	0 (0%)	0 (0%)	0 (0%)	1439 (20%)	

**Table 7:** Use of various GLP-1 RAs according to year in patients older than 46 years (*p*-value for all in-between GLP-1 RA comparisons).

Characteristic	N	Overall N = 17,721	2018 N = 179	2019 N = 411	2020 N = 601	2021 N = 1764	2022 N = 3892	2023 N = 5256	2024 N = 5618	*p*-Value
	17,721									<0.001
Dulaglutide		3179 (18%)	115 (64%)	231 (56%)	195 (32%)	389 (22%)	1148 (29%)	658 (13%)	443 (7.9%)	
Liraglutide		4818 (27%)	64 (36%)	180 (44%)	214 (36%)	552 (31%)	1247 (32%)	1398 (27%)	1163 (21%)	
Semaglutide p.o.		2763 (16%)	0 (0%)	0 (0%)	0 (0%)	0 (0%)	362 (9.3%)	913 (17%)	1488 (26%)	
Semaglutide s.c.		5968 (34%)	0 (0%)	0 (0%)	192 (32%)	823 (47%)	1135 (29%)	2287 (44%)	1531 (27%)	
Tirzepatide		993 (5.6%)	0 (0%)	0 (0%)	0 (0%)	0 (0%)	0 (0%)	0 (0%)	993 (18%)	

**Table 8:** Use of various GLP-1 RAs according to year in obese patients (*p*-value for all in-between GLP-1 RA comparisons).

Characteristic	N	Overall N = 28,545	2018 N = 135	2019 N = 427	2020 N = 741	2021 N = 2471	2022 N = 6315	2023 N = 8503	2024 N = 9953	*p*-Value
	28,545									<0.001
Dulaglutide		3175 (11%)	84 (62%)	226 (53%)	168 (23%)	393 (16%)	1261 (20%)	617 (7.3%)	426 (4.3%)	
Liraglutide		11,160 (39%)	51 (38%)	201 (47%)	353 (48%)	1044 (42%)	2960 (47%)	3492 (41%)	3059 (31%)	
Semaglutide p.o.		4007 (14%)	0 (0%)	0 (0%)	0 (0%)	0 (0%)	526 (8.3%)	1266 (15%)	2215 (22%)	
Semaglutide s.c.		8157 (29%)	0 (0%)	0 (0%)	220 (30%)	1034 (42%)	1568 (25%)	3128 (37%)	2207 (22%)	
Tirzepatide		2046 (7.2%)	0 (0%)	0 (0%)	0 (0%)	0 (0%)	0 (0%)	0 (0%)	2046 (21%)	

## Data Availability

Data are available on reasonable request made to the corresponding author. The data are not publicly available due to institutional policies.

## Abbreviations

The following abbreviations are used in this manuscript:

GLP-1 RA	GLP-1 receptor agonist
GIP	Glucose-dependent insulinotropic polypeptide
FDA	Food and Drug Administration
AHI	Apnoea–hypopnoea index
